# Mapping the Landscape of Surgical Registries in the United Kingdom: A Review According to the SWiM Methodology

**DOI:** 10.29337/ijsp.169

**Published:** 2021-12-31

**Authors:** Connor J. S. Moore, Kerry N. L. Avery, Amber Young, Robert J. Hinchliffe, Xavier L. Griffin, Shelley Potter

**Affiliations:** 1 Bristol Centre for Surgical Research, Bristol Medical School, Department of Population Health Sciences, Canynge Hall, 39 Whatley Road, Bristol, BS8 2PS, United Kingdom; 2 Barts Bone and Joint Health, Barts and The London School of Medicine and Dentistry, Queen Mary University of London, London, UK; 3 Department of Trauma and Orthopaedic Surgery, Barts Health NHS Trust, London, UK; 4 Bristol Breast Care Centre Southmead Hospital, Southmead Road, Westbury-on-Trym, Bristol, BS10 5NB, UK

**Keywords:** Surgical Registries, Registry, Audit Database, Surgical Device monitoring, surgical procedure monitoring, national audit

## Abstract

**Background:**

Well-designed surgical registries are essential for high-quality patient centred evaluation of implantable devices and surgical procedures. The importance of registries was highlighted in the recent Cumberlege report that detailed important innovation failures such as the use of vaginal mesh. Many surgical registries exist, but it is currently unclear how different registries are funded, governed, designed, and how their databases are hosted and utilised. There is therefore a need to understand the variation and characteristics of existing surgical registries to identify limitations and make recommendations for improvement. This work aims to understand the characteristics and heterogeneity in the design, governance, and function of existing surgical registries in the United Kingdom (UK).

**Methods:**

Existing surgical registries will be identified using multiple data sources including surgical society websites; search engine review; a targeted search of the Medline and Embase databases and expert knowledge. The data identified were reviewed following the synthesis without meta-analysis (SWiM) methodology. This information will be gathered from sources in the public domain only to fully understand registry transparency for professionals and the public. Details of each registry including disease area/condition/device evaluated; types of outcomes collected; governance, consent, and oversight; linkage to other datasets and funding will be extracted using a standardised data extraction tool. Characteristics of identified registries will be summarised into a narrative review.

**Dissemination:**

Findings will be presented at national and international conferences and published in peer-reviewed journals. Results will be presented to key stakeholders including surgeons, methodologists, trialists, regulators, data managers and patients to provide an up-to-date description of the current state of surgical registries in the UK. This work will inform a consensus process to agree how the design of new and existing registries can be optimised to support high quality research to benefit patients and the NHS.

**Highlights:**

- Well-designed surgical registries are essential for high-quality patient centred evaluation of implantable devices and surgical procedures
- Presently there is limited understanding on how these registries are designed, governed, what data they collect and how this data is utilised for research.
- This review aims to map the landscape of surgical registries in the UK, and understand how they are optimised for research.

## 1. Introduction

Well-designed surgical registries are essential for high-quality patient centred evaluation of implantable devices and surgical procedures [1,2,3]. The value of registries for patient benefit has been highlighted in orthopaedics with the National Joint Registry demonstrating higher than expected revision rates for metal-on-metal hip implants [4]. Whereas, failure to monitor devices has led to high profile examples of patient harm such as the ‘Poly Implant Prothese breast implant scandal’ [5]. Most recently, the importance of registries and ‘collecting what matters’ and the principles of ‘collect one, use often’ were highlighted in the Cumberlege report that detailed the failings of the introduction and evaluation of vaginal mesh in the United Kingdom (UK) [6].

The US Agency for Healthcare Research and Quality (AHRQ) defines a patient registry as “an organized system that uses observational study methods to collect uniform data to evaluate specified outcomes for a population defined by a particular disease, condition or exposure, and that serves a predetermined scientific, clinical, or policy purposes” [3]. Preliminary work has suggested that there are currently over 100 registries in use in the UK that fit this definition, with around 30 of these being specific to surgery. Significant heterogeneity is likely to exist in surgical registries, given that previous research into non-surgical registries has identified that registries are often designed independently of each other to collect specialty-specific information, do not link with wider NHS data systems, frequently encounter funding issues and suffer from sporadic data entry [7,8].

Well-designed registries have the potential to support high-quality efficient research including registry-based randomised control trials (RCTs) [9,10] that are increasingly utilised to overcome the traditional barriers to RCTs including high cost and complexity. Hypothesised variation in the design of existing surgical registries may limit this potential. For example, it is unclear whether individual registries collect outcomes important to professionals or patients (e.g., patient reported outcome measures (PROMs)) or whether they have the capacity for linkage to other data sets for efficient long-term evaluation. While the characteristics of an ideal surgical registry have been described [1], there is currently no consensus as to how registries may be optimised for research or whether this is of value.

There is a need to understand the current heterogeneity of existing national surgical registries as the first step to establishing consensus as to if and how new and existing registries may be optimised for research. The aim of this study is therefore to describe the design, content, and function of existing national surgical registries in the UK.

## 2. Aim

The aim of this study is to identify, describe and summarise the key characteristics of existing national surgical registries in the UK.

### 2.1 Objectives

1. To identify national surgical registries used in the UK
2. To summarise the characteristics of surgical registries including following:
  - Design
  - Funding
  - Data management, collection & reporting
  - Nature of data collected (e.g., PROMs)
  - Governance including use of patient consent and oversight
  - Linkages to other routinely collected datasets
  - Use in clinical research
3. Summarise the key differences and similarities between registries.
4. Develop a list of key features for inclusion in a future consensus process

## 3. Methods

### 3.1 Search strategy

Existing surgical registries will be identified using the following methods in accordance with the synthesis without meta-analysis (SWiM) methodology [11]:

- Systematic search of all surgical societies’ websites, identification of associated registries, their websites, and their documentation.
- Targeted search of Embase and Medline databases to identify protocols released prior to launch of each registry: key words = surgery; surgical registries; registry; registries; audit; audit database.
- Snowball searching using references of identified papers and reviews
- Clinical expertise and knowledge from research team and experts in registry development

### 3.2 Registry Definition

A registry will be defined as “an organized system that uses observational study methods to collect uniform data to evaluate specified outcomes for a population defined by a particular disease, condition or exposure, and that serves a predetermined scientific, clinical, or policy purposes” [3].

### 3.3 Registry selection

A list of identified registries together with their parent surgical speciality and/or specific intervention/device(s) considered will be developed and reviewed by the study team. Only UK based registries evaluating a surgical intervention(e.g. the National Flap Registry [2]) and/or device (e.g. the National Joint Registry [12]) and/or conditions treated with surgery (e.g. International Burns Registry [13]) will be included in the review. For the purposes of this review, a surgical intervention will be defined as a procedure involving an incision with instruments usually performed in an operating theatre and normally involving anaesthesia and/or respiratory assistance’ [14]. Registries of purely medical conditions (e.g. the National Haemophilia database [15]) will be excluded. A list of all registries encountered will be developed. Details of each registry will then be acquired from registry/surgical society website and associated documents and/or via full-text articles that have described each registry. Whilst unconventional, the systematic search of website information is necessary to fully ascertain registry characteristics that aren’t described in protocols for registry development. Uncertainties will be discussed with the study team and clear reasoning for registry exclusion will be documented.

### 3.4 Data extraction

A data extraction proforma will be developed and iteratively refined by the study team to include all items of potential relevance to registry design; funding; data management, collection, and reporting; nature of data collected (e.g., PROMs); governance including the need for individual patient consent and registry oversight; linkages and any additionally relevant contextual factors; use in research. This will be informed by the literature [1] and clinical and methodological expertise from the study team. The data extraction proforma will be piloted with 8 or more registries and refined as needed prior to commencing full data extraction.

Data sources and associated documentation on registry websites will be utilised in addition to any published literature. This information will be gathered from sources in the public domain only. Data extraction will be performed by one reviewer with approximately 20% of registries double data extracted by an independent second reviewer to ensure methodological rigor. Any areas of disagreement or uncertainty will be resolved by discussion with the wider study team.

### 3.5 Data analysis

A summary of key demographics of each registry will be presented and the results tabulated where relevant. A descriptive analysis of these data will be used to compare characteristics between registries that are utilised for surgical devices and/or procedures. A narrative synthesis will be used to describe the study findings and generate recommendations for best practice.

### 3.6 Assessing bias and meta-bias

This will be assessed as to whether this review is compliant with the SWiM [11] reporting checklist including: risk of bias in study design, risk of bias in individual studies, presenting data on the risk of bias across information encountered.

### 3.7 Limitations of the synthesis

The main limitation of this study is that the study group are assessing information available in the public domain only. This means that some key information on registry characteristics could be missed, by nature of the study design. Where relevant this transparency of information will be discussed keeping in mind the limitations of the study design. It is also possible that some registries may not be included due to being missed in the literature search. Efforts to address this have been made by using a novel review approach which includes search engine review and extraction of data on registry websites. This will address some of the limitations inherent to systematic review of available literature which would miss registries that did not publish a protocol prior to development.

## Research registration number

Name of the registry: N/a

Unique Identifying number or registration ID: N/a

Hyperlink to the registration (must be publicly accessible): N/a.

## Author informations

**Connor Moore:** Conceptualization, Methodology, Formal Analysis, investigation, data curation, writing – original draft, writing – review and editing, visualisation, project administration.

**Kerry Avery:** Conceptualization, methodology, writing – review and editing.

**Amber Young:** Conceptualisation, methodology, writing – review and editing

***Robert Hinchliffe:*** Writing – review and editing, conceptualisation, methodology

**Xavier Griffin:** Supervision, writing – review and editing, methodology

**Shelley Potter:** Conceptualization, methodology, resources, writing - review and editing, supervision, project administration, funding acquisition
